# Can early pregnancy decorin levels be a potential predictor for preterm birth?

**DOI:** 10.12669/pjms.38.8.6551

**Published:** 2022

**Authors:** Levent Ozgen, Gulten Ozgen

**Affiliations:** 1 Levent Ozgen, MD. Gynecological Oncology Surgery, Uludag University Faculty of Medicine, Bursa, Turkey; 2 Gulten Ozgen, MD. Department of Obstetrics and Gynecology, University of Health Sciences, Bursa Yuksek Ihtisas Research and Training Hospital, Bursa, Turkey

**Keywords:** biomarker, decorin, preterm birth, preterm delivery, premature rupture of membranes

## Abstract

**Objective:**

This study investigates the role of maternal decorin levels measured in the early second trimester of pregnancy in detecting the potential for preterm birth in late pregnancy.

**Methods:**

The prospective, case-control study was carried out in tertiary university hospital from June to December 2021.Maternal serum samples were collected from 350 women aged 18–40 years with a singleton pregnancy during early second-trimester screening for aneuploidy, and stored at -80°C. All participants were followed up until delivery and 25 patients diagnosed with preterm birth group and 40 full-term healthy women were included in the study.

**Results:**

The median maternal serum decorin level was 3.82 (1.15–12.37) ng/ml in the preterm birth (PTB) group and 4.63 (1.20–10.02) ng/ml in the control group, there was no statistically difference between the groups (p = 0.111). The mean gestational age was statistically significantly lower in the preterm birth group (33.1±2.7 weeks) than in the control (39±1.16 weeks) (p <0.001). The mean fetal weight was statistically significantly lower in the preterm birth group (2023.8±477 g) than in the control group (3309.7±353 g) (p <0.001).

**Conclusion:**

Early second-trimester serum levels of decorin alone may not be a sufficiently accurate biomarker as a biochemical model for the prediction of preterm birth in asymptomatic women.

## INTRODUCTION

Preterm birth is defined as birth before 37 weeks of gestation. As one of the leading causes of perinatal and neonatal morbidity and mortality in many countries around the world, it is considered a major obstetric and health problem. The prevalence of preterm birth is higher than 11% in many regions, and ranges from 5–7% in northern European countries, indicating clear differences in regional preterm birth rates.^1^,^2^

While various spontaneous or iatrogenic mechanisms can lead to preterm birth, 75% of cases are spontaneous.^3^ Spontaneous preterm birth (SPB) occurs due to the preterm premature rupture of fetal membranes (PPROM) in 30–40% of cases, or following preterm labor caused by uterine contractions that lead to cervical dilation without rupture in approximately half of the cases.^4^

The pathogenesis of preterm birth is still not fully understood, although the results of previous studies indicate a complex syndrome with multiple causes. PPROM, short cervical length at 23–24 weeks of gestation, positivity for fibronectin, and history of PPROM and connective tissue disorders associated with connective tissue damage, such as Ehlers Danlos Syndrome (EDS) that can cause a weakening of fetal membranes, may serve as guides for the early detection of preterm birth.^5^ Reducing preterm births, whether spontaneous or iatrogenic, is extremely important not only in reducing neonatal mortality or morbidity, but also in minimizing the social impact of prematurity. There is, however, currently no serum marker that can diagnose and predict preterm birth, and potentially contribute to reducing its incidence.

Decorin (DCN**)**, a member of the small leucine-rich proteoglycans (SLRPs), is found in the extracellular matrix (ECM). As a molecule that is produced mainly by reproductive tissues, Decorin is, together with biglycan, the main proteoglycan forming the intermediate and reticular layers of human fetal membranes.^6^ It is highly homologous to biglycan, another proteoglycan, and these two proteoglycans contribute to the strength of connective tissues through fibrillogenesis regulation, cell organization, and Type-1 collagen attachment and stabilization in vivo. Decorin has also been shown to affect various cell functions, such as proliferation, propagation, migration and differentiation, and to be one of the physiological regulators of inflammation.^6^

There have been several recent studies investigating the role of decorin in PPROM. Underhill et al. (2019) reported maternal biglycan levels to be high in early pregnancy, while decorin levels were low in the group of pregnant patients who went on to develop PPROM in late pregnancy.^7^

The present study investigates the clinical significance of decorin levels measured in the early second trimester of pregnancy, exploring its potential in the determination of preterm birth developing in late pregnancy.

## METHODS

This single-center, prospective, case-control study was carried out a tertiary university hospital – between June and December 2021. Written informed consent was obtained from each participant prior to their involvement in the study. The study protocol was approved by the hospital’s local Ethics Committee (Date: 2011-KAEK-25 2021/08-03).

In the study, maternal serum samples were collected from 350 women aged 18–40 years with a singleton pregnancy during second-trimester screening for aneuploidy between (15+0–19+6) weeks of gestation. The patients’ medical history, age, height, weight, gravidity and parity, and history of gestational hypertension (GHT), preeclampsia (PE), fetal growth restriction (FGR), PPROM and preterm delivery as adverse pregnancy outcomes were recorded. The pregnancy follow-ups, peripartum outcomes and delivery details of all participants were retrieved from the hospital database systems and the records of the delivery clinics. Our study included a total of 65 patients, including 25 pregnant women diagnosed with preterm birth based on pregnancy and delivery details, and 40 full-term healthy pregnant women with no complications during pregnancy follow-up, delivery or the postpartum period. The patient and control groups were matched for age and BMI.

Preterm birth is defined by the World Health Organization (WHO) as a live birth occurring before 37 weeks of gestation, while PPROM refers to the premature rupture of fetal membranes before the onset of labor.^8^

Pregnant women with multiple pregnancies, incompetent cervix, congenital anomalies, intrauterine growth retardation (IUGR), intrauterine fetal demise or gestational hypertensive diseases, and those using acetylsalicylic acid or anticoagulants were excluded from the study. Other exclusion criteria were pre- and gestational diabetes mellitus, polyhydramnios, connective tissue diseases, vasculitis, chronic kidney and/or liver disease, hyper/hypothyroidism, hematological disorders and smoking, and a previous pregnancy history of chorioamnionitis, PPROM and preterm birth.

Demographic data such as maternal age, BMI (weight in kg/height in m^2^), gravidity and parity were recorded for each patient at the time of sampling. Gestational age was calculated according to the first day of the last menstrual period, and was confirmed by measuring the first-trimester crown-rump length on obstetric ultrasonography.

### Sample collection and analysis:

The venous blood samples collected from all study patients at the early second-trimester screening for aneuploidy (15–20 weeks) were centrifuged at 3500 rpm for 10 minutes and then stored at -80^o^C. Subsequently, the serum decorin levels of the study patients in the Preterm birth and healthy control groups according to pregnancy and delivery outcomes were measured using a *Human Decorin Elisa kit* (China).

### Statistical Analysis:

Data were analyzed by SPSS 22.0 for Windows (SPSS Inc, Chicago, IL, USA) statistics program. Shapiro-wilk test was used to determine the normality of distribution of variables. Descriptive data were expressed as mean ± standard deviation for normally distributed variables while they were expressed as median (minumum-maximum) or non-normally distributed ones. Categorical variables were shown as percentages and frequency. Student t-test was used for variables showing normal distribution between two groups and Mann-Whitney U-test was performed to compared non-normally distributed variables between two groups. Categorical variables were compared between groups by Pearson Chi-square and Fisher’s exact test. Alfa value <0.05 was considered as statistically significant.

## RESULTS

The study, conducted with 350 pregnant women, included 25 patients diagnosed with preterm birth (7.15%) in which preterm delivery occurred following PPROM in 17 cases (68%) and preterm labor in eight cases (32%).

The mean age of the patients was 28.2± 6.6 and 27.7±4.1 years in the preterm birth and control groups, respectively, with no statistical difference between the groups (p = 0.756). The mean body mass index was 28.2±6.1 in the PTB group and 29.2±4.5 kg/m2 in the control group (p = 0.11). There was no statistically significant difference between the two groups. Similarly, there was no difference between the groups median gravidity and parity numbers (respectively p=0.154 and p=0.853). The median gestational age at the time of serum sampling was 16.35 weeks for the preterm birth cases and 16.85 weeks for the controls (p = 0.177). The demographic data of the study participants are presented in Table-I.

**Table-I:** Demographic characteristics of study groups.

	Control (n=40)	Preterm Birth(n=25)	p-value
Age, year	27.7±4.3	28.2±6.6	0.075^b^
BMI, kg/m^2^	29.2 ± 4.5	28.2 ± 6.1	0.1 ^a^
Gravidity(n)	2 (1-4)	2 (1-5)	0.154 ^a^
Parity (n)	1 (0-3)	1(0-3)	0.853 ^a^

Data are given in mean ±SD or median (min-max), unless otherwise stated.

a Mann-Whitney U test.

b Independent samples t-test.

The mean maternal serum level of decorin was 3.82 (1.15–12.37) ng/ml in the PTB group and 4.63 (1.20–10.02) ng/ml in the control group, with no statistically significant difference between the two groups (p = 0.111). There was no significant difference in the decorin levels of the preterm labor and PPROM groups (p=0.344). The mean gestational age at birth was statistically significantly lower in the PTB group (33.1±2.7 weeks) than in the control group (9±1.16 weeks) (p <0.001). The average fetal weight was statistically significantly lower in the PTB group (2023.8±477 g) than in the control group (3309.7±353 g) (p <0.001). Similarly, the one minute and five minutes Apgar scores were lower in the PTB group than in the control group (p <0.001 for both parameters). Clinical characteristics of the study groups are summarized Table-II.

**Table-II:** Clinical characteristics of the study groups.

	Control (n=40)	Preterm Birth (n=25)	p-value
GW at the time serum sample collection, week	16.85±1.96	16.35±1.96	0.170^a^
GW at delivery, week	39±1.16	33.1±2.7	0.001^a^
Birth weight, gr	3309.7±353	2023.8±477	0.001^b^
APGAR score, 1 min	9 (8-9)	8 (0-9)	0.001^a^
APGAR score, 5 min	10 (8-10)	9 (0-10)	0.001^a^
DCN level, ng/mL	4.63 (1.20-10.02)	3.82 (1,15-12,37)	0.111^a^

Data are given in mean ±SD or median (min-max), unless otherwise stated.

a Mann-Whitney U test.

b Independent samples t-test. GW: Gestational Week; DCN: Decorin

While 64% had a Caesarean delivery and 36% had a vaginal delivery in the preterm birth group, the rate of acute fetal distress was 48%. The number of newborns followed up in the neonatal intensive care unit was 13 (52%) (Table-III).

**Table-III:** Distribution of categorical variables according to preterm birth groups.

		Preterm Birth Group	

		n	%
PPROM	No	8	32.0
	Yes	17	68.0
Preterm labor	No	17	68.0
	Yes	8	32.0
AFD	No	13	52.0
	Yes	12	48.0
Type of delivery	Vaginal	9	36.0
	C/S	16	64.0
Neonatal ICU	No	12	48.0
	Yes	13	52.0
Total		25	100.0

Data are given in number and frequency. PPROM: Preterm Premature Rupture of Membranes,

AFD: Acute fetal distress, C/S: Cesarean Section, Neonatal ICU: Neonatal Intensive Unit.

## DISCUSSION

Preterm birth places a great financial burden on society due to the resulting prematurity, and the need for neonatal intensive care and specialized health personnel. The development of supportive therapies and facilities also increases neonatal morbidity, thereby leading to the prolonged life expectancy of newborns and potential comorbidities.^9^ There are yet no tests available for the prediction of all spontaneous preterm birth. The prediction of delivery in women presenting with a threat of preterm birth gives obstetricians time for the in-utero transfer of the fetus to the appropriate hospital through prophylactic interventions such as prenatal tocolysis, corticosteroids and magnesium sulfate. Understanding the physiology and pathology behind full-term and preterm birth can help improve this negative condition.

Preterm birth can occur for various reasons, such as inflammation, hemorrhage, activation of the maternal or fetal hypothalamic-pituitary axis, immune dysregulation, distension of the myometrium and cervical insufficiency.^10^ All such processes may share a common pathway for the commencement of labor, resulting in myometrial contractions, degradation of extracellular matrix components, and the release of mediators inducing inflammation and apoptosis. Although the etiologies are common, spontaneous preterm birth and PPROM originate from different pathophysiological pathways, although inflammation is a common underlying mechanism in both conditions.^11^,^12^.

Decidualization involves extensive changes in the ECM, indicating the transformation of fibroblasts into secretory cells in the endometrial stromal tissue. In pregnancy, this process is associated with the remodeling of the spiral arteries, as well as the transformation of the uterine glands and uterine natural killer (uNK) cell flow, ensuring adequate blood supply to the growing fetus. Decorin is found in the cervix, uterus, fetal mesenchymal cells, and placental decidua in humans at varying levels, depending on gestational age. Decorin binds to many components of the ECM, growth factors and receptor tyrosine kinases, and is required, together with N core proteins, for collagen fibrillogenesis and for increasing fibril diameter and tensile strength.^13^

Decorin also influences the movement of cells by modulating the interaction between membrane surface receptors and matrix protein ligands. While decorin-expressing cells have less migration ability, they also accumulate matrix fibronectin. There have been numerous studies reporting that the amount of both decorin and collagen decreases as the membrane tensile strength reduces before birth in human fetal membranes.

In their model study of mice, Calmus et al. reported biglycan and decorin to be necessary for the maintenance of an uneventful pregnancy until term, and to have a gene dose-dependent and compensatory effect on each other.^14^ This finding is in line with Wu et al.’s suggestion that biglycan/decorin-null fetal membranes show abnormal morphology. Wu et al. reported that decorin signaling supported membrane remodeling in early gestation in a Transforming Growth Factor-β (TGFβ)-dependent manner, and fetal membrane stabilization in late gestation, without any change in TGFβ levels, and that exogenous recombinant decorin rescued components of the TGFβ signaling pathway in fetal membrane mesenchymal cells. While these findings indicate that external modifications of the ECM components allow fetal membrane stabilization. These findings allow the manipulation of ECM components for fetal membrane stability, while also presenting new targets for researches into prevention strategies for PPROM.^15^

The 2014 study by Meinert et al. revealed the role of biglycan and decorin in the maintenance of pregnancy in an inflammation setting, and also in the fetal membrane response to inflammation.^16^

Various studies have shown that decorin concentrations decrease in fetal membranes immediately after a term birth, while the levels decrease in amniotic fluid in preterm and full-term deliveries. This decrease in decorin during the third trimester and delivery may contribute to the mechanical weakening, leading to the onset of labor.^13^,^17^

Horgan et al. reported a significant degradation of decorin in the amnion in chorioamnionitis, and established a decreased fetal membrane decorin expression in preterm birth with PPROM when compared to preterm birth without PPROM only in the presence of infection. The authors suggested that the low-level regulation of decorin due to inflammation might be responsible for the weakened fetal membranes in PPROM.^18^

Atalay et al. found serum decorin levels to be significantly lower in their preterm labor group than in their healthy control group in their study of women with 24–32 weeks of pregnancy presenting with preterm labor. The authors reported that while serum decorin levels alone could predict preterm birth before 37 weeks of gestation, a combined measurement with cervical length was required to predict preterm birth within 7 days and before 34 weeks of gestation.^19^

Underhill et al.’s first study in 2019 reported maternal biglycan levels to be high in the early second trimester, and decorin levels to be low in the group of pregnant patients who went on to develop PPROM in late pregnancy.^7^

A mice model study by Pantham et al. found that the loss of ECM proteins biglycan and decorin led to marked changes in the transcriptomic profile of fetal membranes during gestation, as well as possibly cell cycle suppression and activation of the complement system. This resulted in a lack of proliferation in fetal membrane mesenchymal cells, and ultimately a loss of fetal membrane integrity and premature birth.^20^

In the light of the data provided in the various studies mentioned above, our study followed pregnant women whose maternal serum samples were collected in the early second trimester, and compared the maternal serum decorin levels between selected healthy patients delivered at term and those with a preterm delivery to determine the clinical significance of decorin in the prediction of preterm birth.

We identified a lower DCN level in the preterm birth group (3.75 ng/dl) than in the healthy control group (4.60 ng/dl), although the difference was not statistically significant (p = 0.111). When the preterm births were classified into two subgroups as PPROM and Preterm labor, based on the etiology, no significant difference was identified between the serum decorin levels of the groups (p = 0.344). Similar to the present study, Mennella et al. reported that decorin levels in healthy pregnancies remained stable with a decrease of less than 1% per week throughout the pregnancy, and that serum decorin and biglycan levels did not exhibit abnormal expression patterns in early pregnancy before the onset of preterm labor or PPROM. These findings support the lack of difference in decorin levels between the preterm birth and healthy control groups reported in the present study.^21^

### Limitations:

It is single-center design and the relatively small sample size. The study was further limited by the fact that we were unable to evaluate decorin levels in the weeks of delivery in the PTB and control groups, and that no investigation was made into the presence of infection. Furthermore, cervical length was not measured in the study group patients in the early second trimester when the maternal serum samples were collected, which is another limitation of the study.

## CONCLUSION

Molecular new markers play an important role in research and clinical practice. However, for the prediction of preterm birth in asymptomatic women based on the results of the present study, early second-trimester serum levels of decorin alone may not be considered a valuable biomarker as a biochemical model. That sad, the results still have the potential to open new doors on a new approach to the identification of women at high risk of preterm labor and PPROM, although large, prospective studies will be required to reach definitive conclusions.
